# FastPrEP: a protocol to evaluate uptake, coverage, and effectiveness of a youth-focused, decentralised and differentiated district-wide HIV pre-exposure prophylaxis program

**DOI:** 10.1186/s12889-025-24722-9

**Published:** 2025-10-31

**Authors:** E Rousseau, DJ Davey, LS Fynn, M Wallace, P Macdonald, C Pike, N Mathola, F Little, K Lebelo, LG Bekker

**Affiliations:** Desmond Tutu HIV Centre, University of Cape Town, Cape Town, South Africa

**Keywords:** Pre-exposure prophylaxis (PrEP), Differentiated service delivery (DSD), Integrated sexual reproductive health services (SRHS), Adolescent and young people (AYP), Adolescent girls and young women (AGYW), Pregnant and breastfeeding women (PBFW), Young LGBTQi people, Male sexual partners (MSP), Cost-effectiveness

## Abstract

**Background:**

Adolescents and young people (AYP) are at increased risk of HIV acquisition and onward transmission in South Africa. The benefits of oral pre-exposure prophylaxis (PrEP) are well established, however, epidemic impact depends on access, effective use and scale-up.

**Methods:**

FastPrEP is an implementation science project that aims to scale up oral and novel PrEP modalities through differentiated service delivery to improve uptake and optimal use of PrEP in key populations. Designed to leverage some of the attributes that make fast-food popular such as efficiency, access, variety (choice) and flexibility, FastPrEP aims to further “demedicalise” the buy-in and access to HIV prevention methods. Attracting young people regardless of HIV serostatus, FastPrEP will deliver PrEP as part of integrated sexual and reproductive health (SRH) packages tailored for key youth populations using mobile clinics (*n* = 4) and local government clinics (*n* = 12) as “hubs” for PrEP initiation. These and other community-based outlets such as youth clubs, courier delivery, schools and other youth frequented venues will serve as “spokes” for PrEP maintenance. FastPrEP aims to scale up PrEP in a dense, HIV-burdened, peri-urban community of approximately one million people in Cape Town. We will adopt the RE-AIM framework to evaluate the FastPrEP intervention among diverse AYP aged 15–29 years (targeting approximately 25 000 AYP) and their sexual partners of any age. We will use a phased approach to build the program and evaluate PrEP uptake and persistence in AYP over time.

**Discussion:**

The overall objective is to evaluate whether community-wide, differentiated delivery of PrEP with regard to user choice leads to greater PrEP uptake among sexually active youths who would benefit most from comprehensive HIV protection. Secondary objectives include evaluating the differences in demographic, socio-behavioural, and risk behaviours between PrEP users and non-PrEP users to determine the effectiveness of demand creation strategies and evaluate the utility of different PrEP outlets. FastPrEP will evaluate the scale-up of community-delivered, differentiated PrEP to AYP and their sexual partners, aiming to improve understanding of the differentiated delivery of PrEP services and their impact on PrEP persistence in key populations.

## Background

While combination HIV prevention programmes have contributed to declines in HIV infection, South African youth continue to acquire new HIV infections daily, concentrated in the populations with high vulnerability to HIV infection and poor prevention access, known as key populations [1, 2]. Although significant efforts have been made to reach and offer HIV prevention, testing, and counselling for all adolescent and young people (AYP), including adolescent girls and young women (AGYW) [3–6], male sexual partners (MSP) [7–9], young men who have sex with men (YMSM) [10, 11], pregnant and breastfeeding women (PBFW) [12–14] and to some extent, transgender women (TGW) [15–17], infections in these key populations continue. Besides ongoing high-risk sexual behaviours and sociocultural risk factors, inadequate access to effective HIV prevention slows rates of HIV incidence reduction.

Access to biomedical HIV prevention options such as oral PrEP (emtricitabine and tenofovir) has been limited in the South African public sector until 2022, further exacerbating HIV prevention access barriers [14, 18–21]. The World Health Organisation (WHO) and others have emphasised a shift towards flexible, tailored, people-centred, differentiated service delivery HIV service model, which, especially if community-based, may effectively address some of the complex social and structural determinants of prevention access [1, 22–24]. Well-known models of differentiated care have been adopted in Sub-Saharan African countries, notably in antiretroviral therapy (ART) [25–27]. Adolescent-responsive services and community-based models tailored to hardly-reached key populations have generated acceptability data [23, 24, 26]. Combining both PrEP and ART services is known as “seroneutral” integrated programming and is thought to reduce infection-related stigma and increase efficiencies by linking prevention and treatment services to testing services. Testing provides the gateway to the services, but adequate demand-creation strategies are often under-resourced and overlooked [23, 24, 27–29]. In addition, demand for HIV services can be enhanced and stigma further reduced by co-packaging with other sexual and reproductive needs such as family planning and sexually transmitted infection (STI) screening.

The overall objective of the FastPrEP project is to evaluate if district-wide, differentiated delivery of PrEP leads to greater PrEP access to the resident sexually active youth population. Formative research examined AYP’s preferences for PrEP delivery, demand creation, and persistence support and included a discrete choice experiment (DCE) and several focus group discussions (FGDs) with a purposefully appointed youth reference group (YRG) comprised of representative AYP. The YRG highlighted the value of accessibility and convenience for its community, as well as some groups' dissatisfaction towards government facilities being the only option for accessing such health services. Deriving inspiration from the rapidly growing and popular Fast Food industry in Africa, AYP identified key attributes that could be applied to HIV prevention delivery. The need for youth-appropriate and fun demand creation campaigns to promote awareness and the utilisation of services and the availability of new products and interventions was emphasised, especially in settings where knowledge and health-seeking behaviours are limited [23, 24]. 

The hub-and-spokes model, described below, was created from this formative work and a previous PrEP demonstration project called POWER, which was implemented in two countries and utilised a variety of outlets including mobile clinics, government health facilities, and courier delivery [30–32].

### Project rationale and aims

While various approaches, including community-based service delivery, have been adopted in recent years to improve oral PrEP uptake and continuation, it is not known whether PrEP delivery differentiation will lead to increased PrEP uptake and persistence and there is little evidence comparing the effectiveness of different PrEP delivery platforms [24]. The FastPrEP protocol, described here, is an implementation science project aimed at evaluating the effectiveness of a youth-focused, decentralised, district-wide PrEP programme in Cape Town, South Africa, prioritising efficiency, convenience and access. The FastPrEP project is designed to provide a multitude of options (i.e. choice) regarding PrEP modality and access points or outlets.

Since the initiation of the FastPrEP protocol, newer biomedical PrEP agents have become available in South Africa and will be introduced and offered alongside oral PrEP. The Dapivirine ring, a vaginal PrEP formulation, has been approved by South African Health Products Regulatory Authority (SAHPRA) for use by non-pregnant women 18 years of age and older. In addition, an intramuscular, long-acting injectable PrEP, long-acting Cabotegravir (CAB-LA), has been approved in all people over 35kg [33]. 

Attracting young people regardless of HIV serostatus, FastPrEP aims to deliver PrEP as part of an integrated sexual and reproductive health package tailored for key youth populations, including AGYW, pregnant and breastfeeding women, transgender women (TGW), and young men who have sex with men (YMSM) aged 15–29 years and their male sex partners of any age.

We are applying the REAIM framework to assess AYP’s PrEP delivery platform preferences and patterns of use. The main objective is to evaluate whether the provision of a choice of PrEP (i.e., choosing a product best suited to individual circumstances and allowing for product switching) and diverse delivery outlets (allowing for delivery platform switching) may lead to increased PrEP uptake, persistence and subsequent reduced HIV infections among AYP.

The primary outcomes will be measured as 1) the number of individuals initiating PrEP compared to all who were offered PrEP; 2) duration on PrEP as indicated by PrEP refill visits and self-report; and 3)measurement of new HIV infections by age strata and population in the community after FastPrEP roll out compared to pre-roll out data from routine clinic visits and other HIV testing. Secondary outcomes include proportional uptake of alternative PrEP modalities, modality switching and use of delivery outlet type and PrEP modalities impact on this. A cost-effectiveness analysis of the various PrEP delivery options will be conducted by the Health Economics and Epidemiology Research Office (HE^2^RO) and will be reported elsewhere.

## Methods

### Project design

Using a phased approach, this type 2, hybrid design implementation project will evaluate PrEP uptake, coverage, persistence and impact over time in a single health district. The RE-AIM framework will be used to assess FastPrEP’s reach, effectiveness, adoption, implementation, and maintenance over the project period.

### Setting

This project is being conducted in the Klipfontein/Mitchells Plain (KPMP) health sub-district, in the metro-East area of Cape Town, Western Cape Province, South Africa. This high-density sub-district is a mixture of low to moderate socioeconomic status and informal and low-cost housing. Most residents rely on government provided health care provision from a series of primary care clinics. FastPrEP is concentrating roll-out efforts in areas with the lowest socioeconomic status and highest HIV prevalence.

**Fig. 1:**
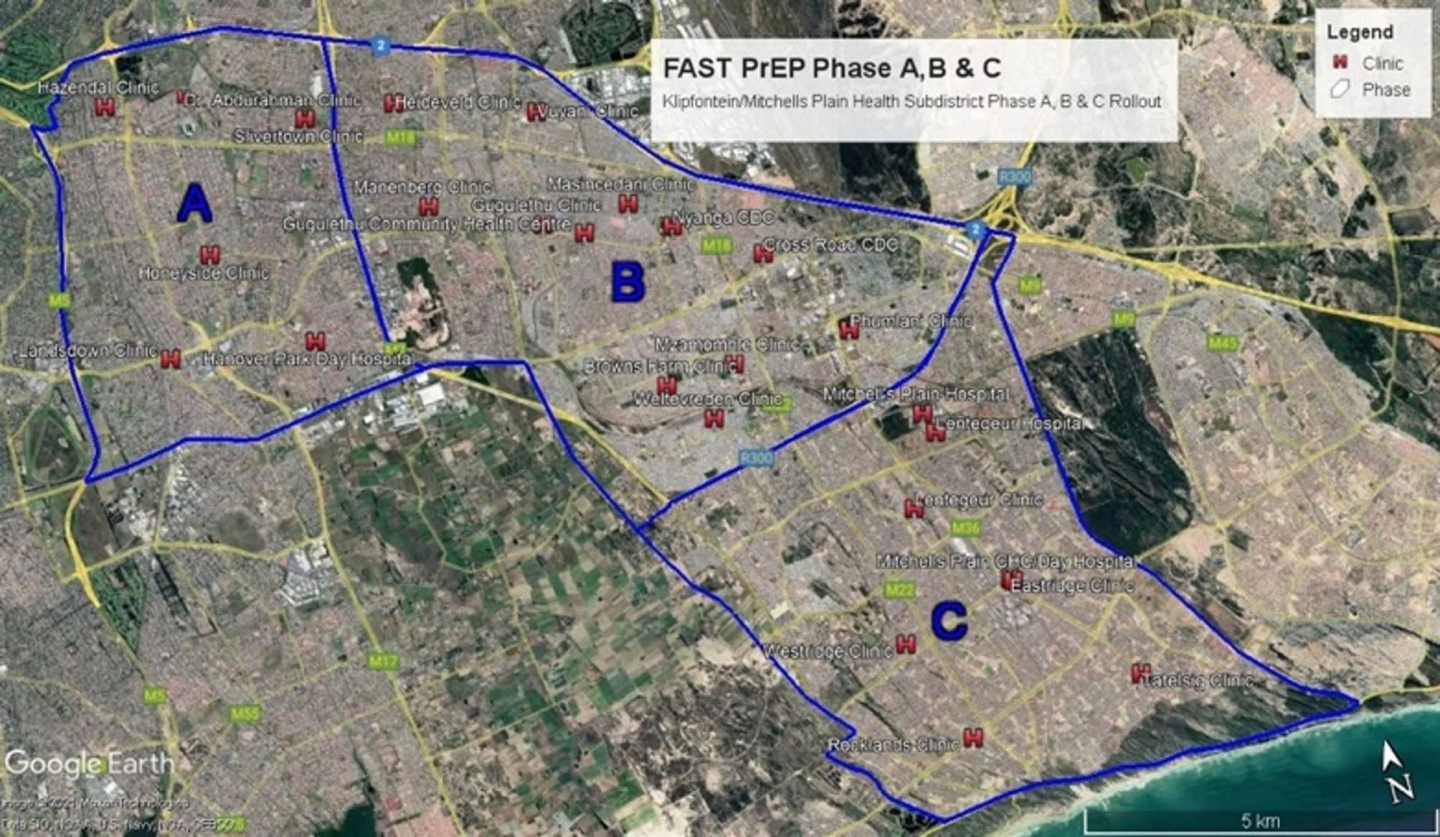
FastPrEP implementation site: Klipfontein-Mitchells Plain sub-district with geographical subsections (A, B, C) for stepped wedge rollout

The selection of community locations and government clinics for FastPrEP delivery was conducted in consultation with the Western Cape Provincial and City of Cape Town municipal health authorities and informed by 2011 census data, HIV prevalence data, and the project team’s existing footprint in the area. To facilitate a phased implementation process, the health subdistrict was divided into three areas: villages A, B, and C (Fig. 1).

Within the public sector, primary care is provided free of charge and includes SRH and HIV services delivered through a nurse-based care system. SRH services include contraception, pregnancy testing, syndromic STI screening and management. HIV services include counselling and testing, treatment, and basic prevention. PrEP has been gradually included in public sector HIV prevention services since 2020 and offered by trained accredited NIMART (“Nurse Initiated Management of Antiretroviral Therapy”) nurses according to SA guidelines [34]. There are 23 primary healthcare facilities in the KPMP sub-district. Services are offered on a first-come, first-served basis and may involve long queues and waiting periods. AYP have described the standard public sector primary health care services as busy, impersonal, prejudicial, stigmatising, fragmented and even hostile [35]. 

### Population

The eligible population comprises HIV seronegative, sexually active cis- and trans- AGYW, PBFW, and MSM aged 15–29 years, and their male sexual partners aged 18 years and older. Eligibility for FastPrEP further includes an interest in HIV prevention and the ability and willingness to provide electronic consent. Clients with pre-existing HIV infection or newly testing HIV-positive are ineligible to join the FastPrEP programme and are referred for immediate treatment and care.

### Intervention

Oral PrEP, available in public clinics since 2020, forms the basis of PrEP standard of care services. Since the implementation of FastPrEP, additional PrEP products have been approved in South Africa, namely vaginal ring (DapiRing) and injectable PrEP (CAB LA). The FastPrEP protocol allows the integration of new PrEP products as they become available, with the expected benefit of gaining early insights into implementation barriers and enablers to the delivery of PrEP choice, within a context where providers and users are less familiar with these products.

**Fig. 2:**
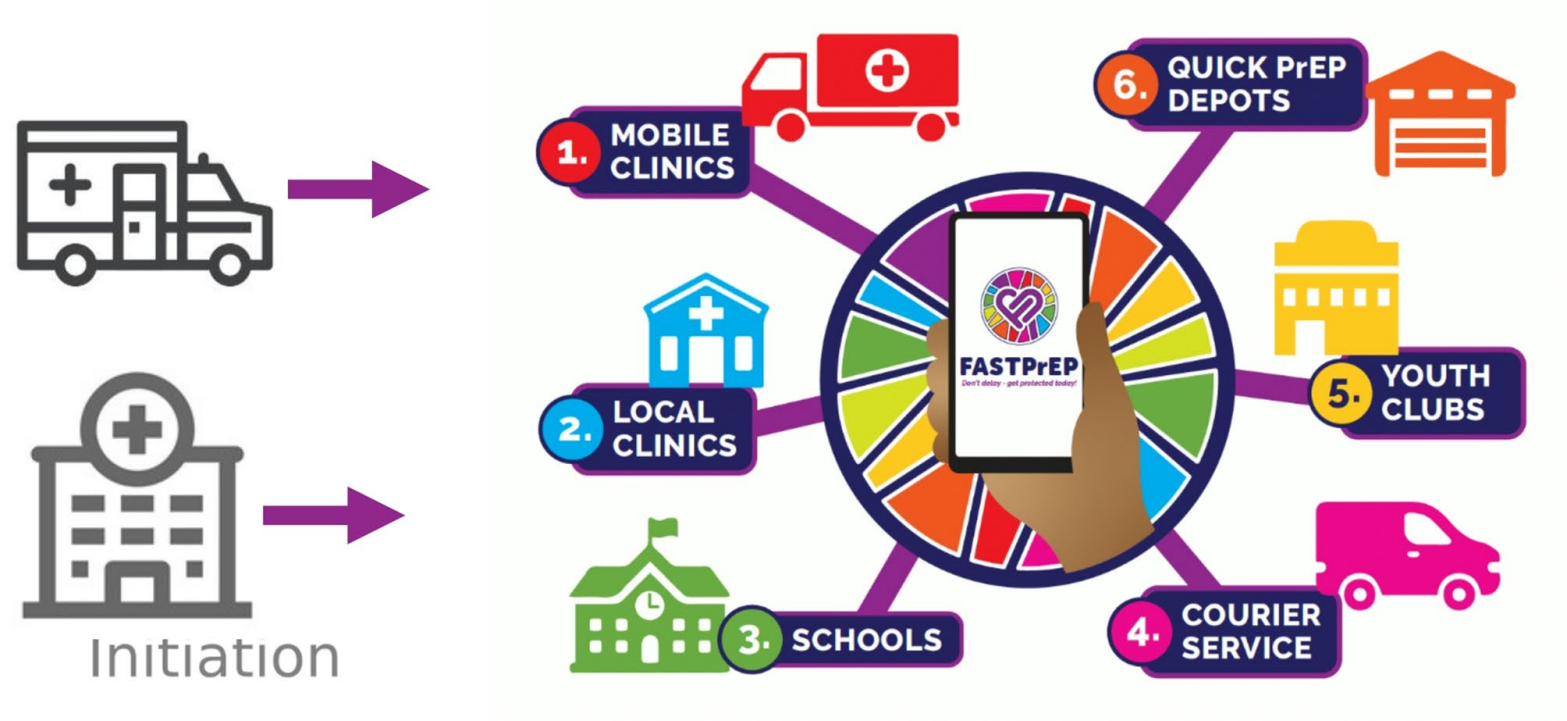
FastPrEP hub-and-spokes model

FastPrEP is scaling up PrEP provision within a broader SRH service package through a hub and spokes service delivery approach over a three-year period (Fig. 2; Table 1) [24, 34]. PrEP users can select their preferred PrEP, access point and delivery method after initiation of PrEP. At each PrEP refill visit, HIV testing and STI screening are conducted, and contraception is offered prior to further prescription. AYP have the option to opt in for an automated WhatsApp message reminder of their follow-up visits.

**Table 1:** Complement of services delivered at each point of the FastPrEP hub-and-spokes model

	HIV testing (counsellor)	HIV self-testing	Pregnancy screening including contraception	Syndromic STI management	STI aetiological test treat	PrEP initiation	PrEP refills	PrEP awareness and demand creation	Peer navigators
Hub									
Mobiles, PHC Clinics	✔ ✔		✔ ✔	✔ ✔	✔	✔ ✔	✔ ✔	✔ ✔	✔ ✔
Spokes									
Schools								✔	
Courier		✔					✔		
Youth Clubs		✔					✔	✔	✔
Quick Depot	✔		✔	✔			✔		

#### Hubs for initiation and maintenance

Local government clinics and community-based mobile clinics are designated “hubs” for PrEP initiation, offering enhanced counselling and information on PrEP as requested. On initiation, participants receive a one-month supply of PrEP, and at their one-month follow-up visit, they are invited to return for three-month refills either at their initiation hub or an alternative “maintenance” outlet (spoke) of their choice.

- *Mobile Clinic PrEP Delivery system.* Four designated mobile clinics are used to deliver PrEP services through an integrated SRH approach. These AYP-tailored mobile clinics (prior to PrEP) have been in service since 2008 to provide SRH and HIV services in locations where AYP congregate. Each mobile clinic is staffed by a nurse, SRH counsellors, an education officer/peer navigator(s), and a driver/security guard. The nurses have medical clinician support telephonically if needed. The clinics provide point-of-care HIV and pregnancy testing and STI testing and treatment using an on-site nucleic acid amplification assay (GeneXpert) and offer a range of hormonal contraception options (oral, injectable and implant).
  Mobile clinics implement a seroneutral approach in which people with a reactive HIV test are provided with appropriate post-test counselling, initiated onto ART, and linked to the nearest clinic for ongoing care. In the FastPrEP project, a peer navigator (trained youth aged 18–29 from the same catchment area) is available to answer any questions about PrEP or ART. Due to their tailored youth SRH services and strategic positioning, the mobile clinics meet many of the “FastPrEP” attributes, such as service efficiency, speed, easy access, and integrated service provision. The FastPrEP mobile clinics’ schedules are posted on social media, which allows clients to know the clinic location for follow-up (or unscheduled) visits.
- *Government facilities as PrEP Delivery platform*. The alternative PrEP initiation hubs are the standard government health facilities in KPMP sub-district. Initially, 12 facilities were selected based on location and readiness to provide PrEP, and two trained FastPrEP peer navigators were allocated to them, whereas counselling and clinical staff are the existing Department of Health (DoH) facility staff. Each facility has undergone a PrEP feasibility assessment to improve PrEP client experience and efficiency. All AYP starting PrEP at a ‘hub’ are informed of the alternative delivery sites available for their PrEP refills. Various in-facility strategies have been provided by the FastPrEP team to make PrEP access more acceptable to AYP and feasible within the government health system context, including:
  i. DOH staff training on PrEP delivery, sensitisation training and mentorship particularly in adolescent-responsive provision of services.
  ii. The use of peer navigators trained at the facilities to optimise clinic flow for efficiency and SRH integration, and.
  iii. Tailoring existing or potential “youth zones” at the clinics on preferred times/days for youth preferential services, designed with the AYP reference group and in collaboration with the respective clinics.

#### Spokes as outlets for PrEP maintenance

For PrEP maintenance, FastPrEP participants can select or switch to their preferred PrEP option, access point and delivery method after initiation of PrEP at an initial FastPrEP hub.

- *Schools*. FastPrEP is targeted towards AYP, many of whom are at secondary school or community tertiary colleges. In FastPrEP, schools (n = 16) are locations for PrEP awareness building, demand creation, and linking AYP to SRH and PrEP services in their areas, especially during weekdays. This includes flyers, talks by peer navigators, education of teachers by FastPrEP nurses, and a map showing the nearest PrEP access site. Mobile clinics are parked near schools and college campuses for PrEP initiation and refills.
- *Courier Delivery Service*. Following PrEP initiation, AYP have the option to request their PrEP refill via courier delivery to their home or other preferred locations by consenting for locator information (address, phone number) to be shared with the courier service. Each participant choosing the courier option is issued a prescription for up to three PrEP refills. Participants are required to attend a physical FastPrEP maintenance spoke every 6 months for a new PrEP script, as per South African health regulations. The medication type is not identifiable on the couriered package and only the identified client can sign for and receive the courier package. In the event of a delivery failure an alternative delivery date or time is arranged and communicated by WhatsApp.
  Upon delivery, the pre-packaged box includes the participant’s PrEP refill and HIV self-testing kit, instructions on how to use the HIV self-testing kit, as well as the referral mechanism should the test result be positive. Participants are required to send a photo of the HIV test result via WhatsApp to a designated research nurse. Failure to do so within 3 days of delivery triggers a follow-up phone call with the participant. In the event of a reactive HIV result, the participant is counselled to halt PrEP use immediately and to visit either the mobile or local government clinic for confirmatory testing where upon ART initiation is advised and provided with a referral for linkage to long-term care.
- *Youth Clubs.* Youth clubs are hosted at safe community-based venues on Saturdays to allow for PrEP access on weekends and within a peer support atmosphere. These events are advertised through WhatsApp groups and other social media platforms. Youth clubs are led by PrEP peer navigators and supported by a nurse for technical assistance and PrEP dispensation.
- *Quick PrEP Depots.* The Quick PrEP Depots are accessible points for obtaining PrEP refills aimed at ensuring swift maintenance visits at smaller mobile trailers staffed with a nurse and counsellor/peer navigator. These depots are placed at a variety of community-based locales in high-traffic areas such as taxi ranks, shops, or hair salons.
- *Private pharmacy outlets.* The role of privately owned community-based pharmacies, many of which run a small adjunct primary health care clinic, as additional PrEP maintenance outlets are also being explored and will be added.

### Demand creation

The FastPrEP implementation strategy is community-driven and youth-led with messaging tailored to specific target populations as part of a demand creation campaign: 1) participatory youth engagement and ongoing consultation with a specifically appointed youth reference group and an existing youth community advisory board; 2) community-wide demand creation awareness campaign including social media and community social media influencers; and 3) youth PrEP champions.

A youth reference group consisting of 80 AYP (ages 15–29) was established during the planning phase of FastPrEP in 2021 to guide, co-create, monitor, and evaluate the implementation of PrEP delivery. The youth PrEP champions are AYP with previous PrEP-use experience who assist in distributing demand creation materials and use in-person and social media platforms to promote PrEP uptake and the SRH services offered by FastPrEP specifically. The micro-influencers are already established in the community and are selected and reimbursed based on their motivation, reach and interest to help promote FastPrEP, HIV prevention, SRH and related topics.

The reach and effectiveness of the FastPrEP demand creation strategies are being evaluated through participant interviews and community surveys to establish, which demand creation methods are most effective in encouraging uptake of PrEP and ongoing use of FastPrEP services.

### Data collection

The RE-AIM framework is being utilised to evaluate the FastPrEP Program [36] and selected measures are summarised in Table 2. All eligible clients are required to register via a digital biometric (finger print) system, give basic demographic data, and e-consent. All PrEP hub and spokes sites are linked online through REDCap to AYP’s biometric data. At the community level, data is entered into REDCap using mobile electronic tablets, automatically synchronising data in near-real time on secure cloud servers. The use of a biometric tracking method has been developed in consultation with the reference groups, and previous projects have shown it to be confidential, feasible, and highly acceptable to ethical boards, participants, and other stakeholders. The personal biometric tracking system is utilised at registration and throughout subsequent FastPrEP platform engagement. This tracks the usage of the different outlets and connects individuals’ medical and pharmacy records to their initiation and follow-up visits. To chart individual PrEP journeys and determine visit frequency, characteristics, delivery preference and persistence, we are monitoring the use of PrEP outlets, bottles/type of PrEP issued, and range of SRH services (i.e. contraception, STI testing, etc.) utilised in all visits.

**Table 2:** RE-AIM approach to FastPrEP evaluation

Variable	Description	Outcomes and indicators	Endpoint and data source
Reach	Types of participants who participate in FastPrEP	● # and % AYP reached by sub-group (AGYW, MSM, MP or PBFW) tested HIV negative and offered PrEP o Numerator: # of PrEP initiators and continuers (1m, 3m, 6m) o Denominator: # of people who test HIV neg who are offered PrEP *by modality * ● Evaluation of the barriers and facilitators to continued and effective use of PrEP product (reasons for adherence/non-adherence, reasons for pauses and discontinuation in sub-set of PrEP users by group in surveys and in-depth interviews (IDIs)	Number and proportion of target audience with PrEP access among at-risk, sexually active population Source: FastPrEP data collection (RedCap) and survey data from participants (sexual activity) and IDIs to identify barriers/facilitators
Effectiveness	Expected or perceived outcomes related to clinical outcomes	● Does the intervention reduce HIV incidence in a sub-study of HIV recency in all those diagnosed with HIV comparing sub-district A, B and C? ● Assessment of negative consequences of PrEP use in AYP (i.e., IPV, stigma) as reported by people on PrEP ● What combination of delivery approaches achieve best PrEP persistence for AYP during periods of sexual activity? ● What are the conditions for AYP’s use of different PrEP delivery sites?	HIV incidence at baseline (before project) and after project in KMP district among target audience (AGYW, MP and MSM) Source: Review of Cape Town Metro HIV testing data by gender/age Negative consequences: review of survey data from FP participants
Adoption	Willingness to use and adoption or non-adoption of interventions	● How representative are the clinics that adopt PrEP successfully vs others? Type and size of health facility vs type of community venue ● Assess representativeness of mobile facilities and service providers that are most effective at PrEP initiation and continuation compared to those with lower PrEP initiates and continuers	Review of clinic data and mobile data from FP team surveys Assessment of adoption and clinical sustainment capacity of mobiles and government facilities: CSAT – clinical sustainment assessment tool survey(39) Source: FastPrEP data collection (RedCap) and survey data from participants and implementers (healthcare providers)
Implementation	Adherence to protocol for implementation and interventions	● Evaluate the various barriers and facilitators at each sites for PrEP uptake and continuation via surveys and in-depth interviews with a subset of implementers and users. ● Evaluate preferences for packaging, delivery of various PrEP modalities via in-depth interviews ● Evaluate modifications that were made to the intervention and why they occurred	Understanding barriers and facilitators to PrEP integration from provider and participant standpoints Source: FastPrEP data collection (RedCap) and survey data from participants (sexual activity) and IDIs with providers and participants to identify barriers/facilitators Modifications reviewed from project reports and logs of modifications made
Maintenance	Participant maintenance behaviours and/or evidence of partners sustainability	● % of AYP by sub-group who continue on PrEP at 3m or 6m ● % of AYP by sub-group who adhere to PrEP while sexually active ● % of coverage of at-risk populations ● % coverage of at-risk sexual events ● Extent to which the model is replicated by DOH/partners in other facilities and Districts	Number and proportion of target audience who continue on PrEP at 3m and 6m among at-risk, sexually active population Review of PrEP provision in other services at 6m post implementation Cost-effectiveness evaluation report Source: FastPrEP data collection (RedCap) and survey data from participants (sexual activity) and partner survey at endline

### Data analysis

We are using a mixed-method approach to evaluate the FastPrEP program. Demographic, behavioural, and clinical characteristics of all AYP enrolled on FastPrEP will be described and compared at baseline. Logistic regression models will be applied to explore factors associated with PrEP delivery platform/outlet engagement for uptake and continued PrEP use. Continued PrEP use will be ascertained at cross-sectional time points. Multinomial logistic regression models will be used where more than two platforms or outlets are available. These models will be adjusted for any confounding characteristics. Survival analysis, including Cox proportional hazard models, will be applied to explore PrEP persistence, adjusting for time-invariant and time-varying confounders. The longitudinal profiles of PrEP use will be explored using group-based trajectory modelling to identify patterns of PrEP use. The pattern of switching modalities will be summarised using cross-tabulations and illustrated using Sankey plots.

Implementation outcomes will be analysed and interpreted within the RE-AIM framework, and qualitative data will be utilised to understand and explain the outcomes of selected RE-AIM dimensions and trends of results across the different hubs and spokes. The qualitative investigation aims to uncover factors influencing PrEP uptake across various delivery sites and explore enablers and barriers to sustained PrEP use, including use patterns and product switches.

Additionally, the project will evaluate the use of newer PrEP agents on PrEP continuation and examine risk behaviours, attitudes, and knowledge gaps among non-PrEP users. We will analyse the qualitative data (i.e., individual and provider interviews and observation reports) using an inductive content analysis technique that includes an iterative coding process and category development utilising qualitative analysis software.

### Ethical considerations

Ethics approval for the FastPrEP project has been granted by the Human Research Ethics Committee (HREC REF# 713/2021) from the University of Cape Town. Participation in the project is voluntary. Participants are asked to provide digital informed consent for data collection, with parental waiver of consent to participate approved for adolescents aged 15–17 years. The provision of services does not require consent, and no young person or male partner is denied services when withholding consent for data collection.

### Dissemination plan

The applied RE-AIM model will inform the development of manuscripts for peer-review publication, presentations at relevant conferences and workshops, briefing reports and identifying relevant target audiences. Project findings will be disseminated to participants, health care professionals, the youth reference group and youth CAB, and other stakeholders through a community-wide demand creation awareness campaign as and when appropriate. In addition, and in collaboration with HE^2^RO, the effectiveness, acceptability, feasibility, cost-effectiveness and affordability analysis will be shared with provincial and national health authorities and other interested agencies and organisations to inform future HIV prevention strategies and optimise national PrEP rollout.

## Discussion

To realise the full benefit of PrEP for HIV prevention, we will need to effectively provide tailored, person-centred PrEP that is offered freely, efficiently, without barriers and at scale. How to achieve this feasibly in resource-constrained environments is not fully understood. Yet, there is increasing evidence that providing differentiated services that take into account different population groups (gender, sex, age differences) and recognise differentiated service delivery platforms and outlets will increase PrEP uptake and persistent use by these diverse populations [24, 35]. FastPrEP was developed to test implementation strategies to improve the quality and efficiency of differentiated PrEP delivery in South Africa at scale and to reach those most at need of accessible, easy and fast PrEP delivery. The program will contribute to our knowledge regarding the feasibility, impact and cost of a community- and facility-based hub and spokes program within Southern Africa. Using the RE-AIM framework, we will systematically identify facilitators, challenges, and opportunities to inform governments and apply the lessons learned to inform policy in the planning, implementing and delivering PrEP as HIV prevention in this region. FastPrEP will demonstrate whether greater participation and persistence are enhanced by removing access barriers and [37] providing choice for South African youth.

FastPrEP is an implementation science project allowing flexibility to adapt and modify the program as lessons are learnt to resolve redundancies and make necessary changes in the scale-up process. However, evaluating the program at scale in a single health district with no matched contemporaneous control population is a limitation. To mitigate this limitation, we will utilise data from other HIV testing and programs within the health district, both historically and contemporaneously, as comparative data and to provide context. Further, generalizability to other areas with different demographics may be limited. We have, however, included diversity of gender, sex and age in our program and are taking it to significant scale over a large health sub-district. The FastPrEP project has also taken a community-wide approach to inform its design, co-creation and implementation with the input from a youth reference group, LGBTQI community advisory board, and other community and government stakeholders involved in overseeing the rollout of PrEP in this community. Finally, FastPrEP is being implemented with frequent and consistent collaborative engagements with key government health stakeholders to ensure that we are creating a feasible and acceptable model for implementation in public health settings. The cost-effectiveness study will help inform affordability. Final programmatic results from the FastPrEP study are expected in 2025, although lessons will be disseminated as they are learned.

## Data Availability

No datasets were generated or analysed during the current study.

## Abbreviations

ART: Antiretroviral Treatment
AGYW: Adolescent Girls and Young Women
AYP: Adolescent and Young People
DCE: Discreet Choice Experiment
DOH: Department of Health
DSD: Differentiated Service Delivery
HREC: Human Research Ethics Committee
KPMP: Klipfontein Mitchells Plain
LGBTQI: Lesbian Gay Bisexual Trans Queer Intersex
MSP: Male Sexual Partners
NIMART: Nurse Initiated Management of Antiretroviral Treatment
PBFW: Pregnant or Breastfeeding Women
POWER: Prevention options for evaluation research
PrEP: Pre-exposure Prophylaxis
REAIM: Reach Effectiveness Adoption Intervention Maintenance
SRH: Sexual and Reproductive Health
STI: Sexually Transmitted Infection
TGW: Transgender Women
WHO: World Health Organisation
YMSM: Young Men who have Sex with Men
YRG: Youth Reference Group
